# Baseline psychosocial predictors of survival in localised breast cancer

**DOI:** 10.1038/sj.bjc.6603091

**Published:** 2006-05-02

**Authors:** U-S Lehto, M Ojanen, T Dyba, A Aromaa, P Kellokumpu-Lehtinen

**Affiliations:** 1 Department of Oncology, Medical School, University of Tampere, FIN-33014 Helsinki, Finland; 2 Department of Psychology, University of Tampere, FIN-33014 Helsinki, Finland; 3 Finnish Cancer Registry, Liisankatu 21 B, FIN-00170 Helsinki, Finland; 4 Department of Health and Functional Capacity, National Public Health Institute, Mannerheimintie 166, FIN-00300 Helsinki, Finland; 5 Department of Oncology, Tampere University Hospital, FIN-36280 Pikonlinna, Finland

**Keywords:** anger, breast cancer, expressed emotion, minimising (denial), repression, survival

## Abstract

Despite the large number of studies on the impact of psychosocial factors on breast cancer progression, there is no certainty about the contributing factors or processes involved. We investigated the relative impacts of socioeconomic, psychological, and psychosocial factors on survival in breast cancer. A consecutive sample of 102 patients (participation 82%) under 72 years of age with locoregional breast cancer completed validated questionnaires on coping with cancer, emotional expression (anger), perceived available support, noncancer life stresses, and quality of life 3−4 months after diagnosis. Survival times were measured from the date of diagnosis to the date of relapse and further to the date of death or date of last follow-up. Cumulative Cox regression analyses were carried out. After controlling for biological prognostic factors, age, and baseline treatment, longer survival was predicted by a long education and a minimising-related coping, while shorter survival was predicted by emotional defensiveness (antiemotionality), behavioural-escape coping, and a high level of perceived support. A shorter event-free time was also predicted by unemployment and depressive symptoms. Cancer survival is affected by a complex combination of psychosocial factors, among which minimising predicts a favourable prognosis and anger nonexpression and escape behaviour an unfavourable prognosis. Higher socioeconomic status is associated with longer survival. High scores in well-being scales may reflect emotional nonexpression.

The impact of psychosocial factors on cancer progression has been investigated in a number of studies (Greer *et al*, 1979, 1990; Pettingale *et al*, 1985; Gross, 1989; Spiegel *et al*, 1989; Forsen, 1991; Barraclough *et al*, 1992; Blanchard *et al*, 1995; Maunsell *et al*, 1995; Dolbeaut *et al*, 1999; Watson *et al*, 1999; Butow *et al*, 2000; Reynolds *et al*, 2000; Spiegel, 2001; Petticrew *et al*, 2002; Garssen, 2004) and many of these have dealt with breast cancer (Greer *et al*, 1979, 1990; Pettingale *et al*, 1985; Spiegel *et al*, 1989; Forsen, 1991; Barraclough *et al*, 1992; Maunsell *et al*, 1995; Watson *et al*, 1999; Butow *et al*, 2000; Reynolds *et al*, 2000; Garssen, 2004). However, the contributing factors are uncertain, and there is also a lack of understanding of the psychological processes and the psychobiological mechanisms involved (Garssen and Goodkin, 1999). In many studies, the theoretical basis has been insufficient, the psychological constructs investigated have varied study by study, and there has been no agreement on definition, operationalisation, and methodology (Butow *et al*, 1999; Garssen and Goodkin, 1999; Reynolds *et al*, 2000; Garssen, 2004; Garssen and Remie, 2004). This has made it difficult to summarise the results and has often led to questionable comparisons of studies evaluating different concepts. Many earlier studies have not controlled for biological prognostic factors or considered socioeconomic status (Garssen and Goodkin, 1999; Garssen and Remie, 2004). Furthermore, the impact of a certain psychosocial factor or factors has often been examined separately (Barraclough *et al*, 1992; Petticrew *et al*, 2002), while possible simultaneous effects have been neglected (Garssen and Goodkin, 1999; Garssen, 2004; Garssen and Remie, 2004).

Current research suggests that the most important psychosocial risk factors in cancer progression may include nonexpression of negative emotions (Garssen and Goodkin, 1999; Reynolds *et al*, 2000; Garssen, 2004) and helplessness/depression (Watson *et al*, 1999), and that life stresses (stressful life events) and low levels of social support may also have an effect (Maunsell *et al*, 1995; Garssen and Goodkin, 1999; Garssen, 2004). Favourable prognoses seem to be predicted by a response pattern of denying and/or minimising the fact of having cancer (Butow *et al*, 1999, 2000; Garssen, 2004). Classical findings concerning concepts like fighting spirit, stoic acceptance, or fatalism (Greer *et al*, 1979; Pettingale *et al*, 1985) have not been replicated in recent studies (Watson *et al*, 1999; Petticrew *et al*, 2002; Garssen, 2004). Psychosocial interventions are thought to have an effect on the mediating psychosocial factors and the well-being and survival outcomes (Fawzy *et al*, 1993; Dolbeaut *et al*, 1999; Fawzy, 1999). The most important psychological factors and processes that influence the psychobiological mechanisms must be identified in order to pinpoint targets for comprehensive cancer care and psychosocial interventions.

The psychobiological mechanisms that affect cancer progression are thought to be related to psychological stress (Kiecolt-Glaser and Glaser, 1999). It has been suggested that the processes of psychological stress include cancer and treatment as independent factors, and that sociodemographic factors, coping, adaptation, personality factors, medical factors, socio-environmental factors, and life stresses are mediators and/or moderators of the processes (Dolbeaut *et al*, 1999; Lehto *et al*, 2005). It is suggested that these affect both quality of life (QOL) and survival. Coping can be seen as the main mediator in the process from cancer and its treatment to the health outcomes (Folkman *et al*, 1986a, 1986b; Holahan and Moos, 1986; Folkman *et al*, 1991; Lazarus, 1993; Reynolds *et al*, 2000; Petticrew *et al*, 2002), and social support and personality factors are assumed to modify the process (Cobb, 1976; Cohen and Wills, 1985; Holahan and Moos, 1986; Burgess, 1987; Temoshok, 1987; Folkman *et al*, 1991; Blanchard *et al*, 1995). Also, the presence of other (noncancer) life events at the time of the illness has an effect on the cancer-related stress processes because patients often need to make efforts in order to cope with them, and this may influence or interfere with coping with cancer or affect the health outcomes (Dolbeaut *et al*, 1999). We have recently presented a model of these factors and their influence on cancer (Lehto *et al*, 2005), and applied it for studying survival in localised melanoma (Lehto *et al*, 2006).

Personality influences coping processes (Greer and Watson, 1985; Temoshok, 1987; Gross, 1989; Dolbeaut *et al*, 1999). A concept of Type C behaviour, a personality characteristic which can tolerate less stress and is thus more vulnerable, is thought to be associated with the progression of cancer (Greer and Watson, 1985; Burgess, 1987; Temoshok, 1987; Gross, 1989; Eysenck, 1994; Garssen and Remie, 2004). The Type C response style is a multidimensional construct which includes nonexpression of negative emotions as a core element, to which are added the dimensions of helplessness and hopelessness in stressful situations and the element of being in behaviour in relation to other people self-sacrificing, over-cooperative, sociable and appeasing, and compliant with external authorities. Stressful situations may be more threatening for people with Type C, because they cannot allow themselves to express negative emotions. It has been claimed that this contributes to less effective coping (Petticrew *et al*, 2002) or a worse outcome. Also, the antiemotionality trait (emotional defensiveness) is related to suppression and control of emotions (Swan *et al*, 1992), and has been reported to have an unfavourable effect on cancer progression (Grossarth-Maticek *et al*, 1985; van der Ploeg *et al*, 1989). It refers to a tendency to avoid emotions related to other people and to exhibit more anger control and less anger expression, and is the opposite of Type A behaviour (Swan *et al*, 1992). Antiemotionality trait is close to the concept of personal defensiveness involved in the nonexpression of negative emotions (Garssen and Remie, 2004) and it results in sociable and appeasing behaviour. These are both included also in the Type C style (Greer and Watson, 1985; Temoshok, 1987; Gross, 1989).

In accordance with our theoretical model (Lehto *et al*, 2005), we assume that the biopsychosocial outcome in cancer is influenced by coping with cancer, which is modified by social support, personality factors, and noncancer life stress, and initiated by cancer-related stressors. Here, we investigate the impact of psychosocial factors on biological (survival) outcomes. Our aim is to investigate the impact of the baseline (3–4 months from diagnosis) mediating psychosocial factors: coping, social support (only perceived available support), nonexpression of emotions (anger), noncancer life stresses, and domains of QOL on survival in locoregional breast cancer.

## MATERIALS AND METHODS

### Patients

Newly diagnosed 30–70-year-old breast cancer patients with localised or regional disease who were admitted for treatment and/or follow-up to the Tampere University Hospital (Finland) Oncology Clinic from January to October 1996 were consecutively included, as described in detail in our previous work (Lehto-Järnstedt, 2000; Lehto *et al*, 2005). *In situ* breast cancers were excluded. Two patients were excluded because of chronic schizophrenia and consequent difficulty in understanding the nature of their disease or treatment and two patients for having had cancer previously, which was thought to have influenced the psychological stress processes. The remainder was invited to participate in the study, firstly by letter and later by personal contact. In all, 82% (*n*=102) of the patients invited participated.

After the exclusion of one male patient, the final study group for the survival analyses consisted of 101 patients, of whom 33 had lymph node metastases. Patients were treated according to national Finnish guidelines for breast cancer treatment. Prior to the interview there was no new antidepressive treatments, no visits to the outpatient psychiatric clinic, and no hospitalisations for psychiatric reasons. The disease and treatment variables of patients are detailed in Table 1, and sociodemographic and socioeconomic variables in Table 2. At 15 February 2005, 31 patients had relapsed and 20 had died.

### Procedure

The patients were interviewed 3–4 months after diagnosis according to a specific structural format and by the same psychologist (the first author). The interviewees completed several structured validated questionnaires, indicating the presence, frequency, or intensity of coping with cancer, emotional expression-related personality factors, perceived available support, noncancer life stresses, and QOL.

Coping with cancer was measured with the Ways of Coping Questionnaire (WOC) (Folkman and Lazarus, 1988; Lazarus, 1993), developed ‘to identify the thoughts and actions an individual has used to cope with a specific stressful encounter’ (here any aspect of breast cancer) using an item structure proposed to form a WOC-CA cancer-specific scale (Dunkel-Schetter *et al*, 1992; Stanton and Snider, 1993) comprising the coping patterns Focusing on the Positive, Distancing, Seeking and Using Social Support, Cognitive Escape-Avoidance, and Behavioural Escape-Avoidance.

The patients' evaluation of the social support which they perceived would be available if needed was measured with the MOS Social Support Survey (Sherbourne and Stewart, 1991; Aalto *et al*, 1995), a 20-item scale for adult patients with chronic conditions divided into perceived emotional/informational support, practical support, and love.

Traits of anger expression were measured with the Anger Expression Scale (AX/Scale) (Spielberger *et al*, 1988; Spielberger and Sydeman, 1994) (24 items) referring to ‘the extent that an individual engages in aggressive behaviour when motivated by angry feelings’ and tapping three dimensions: Anger-in (angry feelings are experienced but held in, ‘repression’), Anger-out (…are expressed in aggressive behaviour), and Anger Control (the outward expression is controlled) (Spielberger *et al*, 1999). Emotional expression was measured with Rational/Emotional Defensiveness (R/ED) (Spielberger, 1988; Swan *et al*, 1992; Fernandez-Ballesteros *et al*, 1997) and Need for Harmony (N/H) (Fernandez-Ballesteros *et al*, 1998) Scales. In R/ED, rationality (R) refers to the extent an individual uses reason and logic as a general approach to coping with the environment (control of anxiety), and emotional defensiveness (ED) (antiemotionality) the extent an individual uses reason and logic to avoid emotions related to other people (to overcome emotional feelings, control of anger).

Stressful life events were evaluated from the year preceding the interview by the Life Experience Survey (LES) (Sarason *et al*, 1978). The more persistent stressful conditions were evaluated by the Chronic Strains Survey (CSS) (13 items, by authors) (Lehto-Järnstedt, 2000; Lehto *et al*, 2005).

The Rotterdam Symptom Checklist (RSCL) (deHaes *et al*, 1990) was used to measure the patients' symptoms (psychological and physical) and their intensity and also perceived quality of life (a single-item index). Depressive symptoms were measured with a 10-item Depression Scale (DEPS) (Salokangas *et al*, 1996), concerning the feelings and symptoms experienced during the previous month and developed for screening of depression in Finnish primary health care settings. The DEPS also includes one item which evaluates hopelessness. Breast cancer-specific symptoms were evaluated with EORTC-breast 23.

Information on the disease and its treatment was collected from hospital records. The ethical committee of Tampere University Hospital approved the research protocol. The researcher was bound by national (The Union of Finnish Psychologists) and international (American Psychological Association) ethical codes of psychology.

### Statistical analysis

Survival was measured from the date of diagnosis (date of the PAD) to the date of advancement of the disease (event-free survival) or date of death (overall survival) or was censored at the date of last follow-up (15 February 2005) for surviving patients. Descriptive statistics, ANOVA, and Pearson's correlation (*r*) were used to describe the sample and regression analysis to investigate the association between the predictive psychosocial factors. The Cox proportional hazards regression model (Cox, 1972) was used to determine the simultaneous contribution of the psychological and psychosocial predictors of survival times controlled for age, biological prognostic factors, and cancer treatment. The proportional hazard assumption was tested (Schoenfeld, 1982) for specific variables and globally.

## RESULTS

### Differences in the psychosocial variables

Chemotherapy was more common in younger patients (*P*<0.001).

Distancing coping was associated with higher age, higher self-reported QOL, less symptoms (*P*-values <0.05), and operation as the only treatment (*P*=0.01). Seeking and using social support was applied more by patients with lymph node metastases, chemotherapy, and any adjuvant treatment (*P*-values <0.05). Cognitive Escape-Avoidance was associated with hormonal therapy and lower self-reported QOL (*P*-values <0.05), and especially with psychological (*P*=0.004) and depressive symptoms (*P*=0.001). Behavioural Escape-Avoidance was associated with higher scores in the Anger-in trait (*P*<0.05). The coping patterns did not vary depending on socioeconomic status.

The scores in the perceived social support scales were otherwise normally distributed, but 15% of patients in emotional/informational support, 33% in practical support, and 44% in love support scored the maximum amount of available support (12% in total score). There were no differences in these scales between disease variables and treatment. The scores increased by family income and amount of emotional/informational support decreased with age (*P*-values <0.05). The patients with high or maximum perceived support scores scored lower in the Anger-in trait (in total scores *P*-values <0.05, in maximum score *P*=0.001) and tended to report a better QOL and less physical symptoms (*P*-values <0.05).

The anger expression traits did not differ by age or disease and treatment. Anger-out and Anger Control traits were associated highly negatively (*r*=−0.58, *P*<0.001), as reported before (Spielberger *et al*, 1988). Emotional defensiveness was positively associated with Anger Control (*r*=0.34, *P*<0.001) and negatively with Anger-out (−0.26, *P*=0.01). The Rationality scale increased with more education (*P*<0.05). Emotional defensiveness was higher among the highly educated compared with the rest (*P*<0.05).

The reported symptoms and QOL (RSCL) did not differ by disease, treatment, or socioeconomic variables, except that patients who were employed experienced less physical symptoms. Depressive symptoms (DEPS) were less common in patients with a longer education (*P*<0.05), but did not vary depending on disease or treatment. Almost one-half (42%) of the patients reported hopelessness.

The only difference that even approached statistical significance between the patients who died and those who were alive at the end of the follow-up was that emotional defensiveness was higher among the deceased (*P*=0.063).

### Predictors of survival and event-free time

In the Cox proportional hazards regression model, we used age, nodus (number of metastatic lymph nodes/the examined), tumour size (mm), Grade 3 in ductal carcinoma (yes/no), hormonal receptors (yes/no), oestrogen receptors (yes/no), type of baseline surgery (breast conserving/mastectomy), baseline adjuvant treatments (radiotherapy, chemotherapy, hormonal therapy), level of education (basic, medium, high), working status (no/yes), family income, and domains of the mediating psychosocial factors, that is, coping with cancer, perceived social support, anger expression, emotional expression, noncancer life stress, and domains of QOL, as dependent variables (as described above using various codings).

Predictors of overall survival were investigated step by step by adding groups of potential prognostic factors; the models for overall survival are detailed in Table 3. Firstly, the biological variables were modelled alone (model 1 in Table 3), then the cancer treatment was added (model 2). Next, we added socioeconomic factors (model 3), and, finally, the psychosocial ones were added (models 4–7). The analyses were continued with only the socioeconomic and psychosocial variables having an important impact (*P*<0.1), therefore Tables 3, 4, and 5 include only these variables.

After adjusting for age and biological prognostic factors, chemotherapy and a high level of education (college or higher) were found to be protective factors. Psychosocial risk factors comprised emotional defensiveness^a^ (antiemotionality), Behavioural Escape-Avoidance^b^ coping, and a high level (over 70 in the range 0–80) of perceived social support (When perceived support was applied as scale variables: MOS emotional/informational support was predictive at *P*=0.056, practical support *P*=0.026, and love *P*=0.055 (total score *P*=0.020)), whereas Distancing^c^ coping was a protective factor. In addition, the depressive symptoms had a survival-decreasing effect (*P*<0.050) before the coping patterns were added into the final models.

To further adjust for the severity of the breast cancer, the variables in model 7 (Table 3) were investigated in patients with no local metastases or chemotherapy (11 deaths), and the effects were found to be about the same (Table 4). When the variables were tested in patients with Gradus <3 only, the effects were similar except that Distancing showed a reduced effect (*P*=0.125).

When the variables were tested one-by-one with the biological and treatment variables (univariate analyses), only emotional defensiveness (*P*=0.007) and Behavioural Escape-Avoidance (*P*=0.057) were significant alone, that is, Distancing coping and level of perceived support were not significant when tested without the emotional defensiveness factor.

When mutual associations between the significant predicting variables were analysed with regression analysis, depressive symptoms were predicted by a stronger Anger-in trait, lower level of perceived emotional/informational support, presence of adjuvant treatment, and lower education. The level of perceived support was increased by lower Anger-in, working outside the home, and a higher level of emotional defensiveness.

When event-free survival (time without relapse) was studied (Table 5), younger age predicted a shorter event-free time (*P*<0.1) along with the biological prognostic factors (nodus, gradus 3). Longer event-free time was predicted by baseline chemotherapy. The socioeconomic factors had an effect only in combination with the psychosocial ones: a long education slightly predicted a longer event-free time (ns), while being unemployed predicted a shorter time without relapse (*P*<0.1). Unlike overall survival, shorter event-free time was only slightly predicted by emotional defensiveness (*P*=0.1). When all the previous variables were included in the model, depressive symptoms and high perceived support tended to predict a shorter time without relapse.

The test for the final models (in Tables 3, 4 and 5) showed that the proportional hazard assumption was not violated either for specific variables or globally, showing that it holds for the chosen models.

## DISCUSSION

Multiple cumulative regression models were applied and the biological prognostic factors were carefully adjusted to investigate the mutual additional effects of treatment, socioeconomic factors, and psychosocial factors on survival in locoregional breast cancer. Along with chemotherapy, a long education and responding to having cancer with distancing/minimising acted as protective factors, while emotional defensiveness (antiemotionality), escape coping, and a high level of perceived social support acted as risk factors.

We chose to study patients with localised breast cancer firstly because it has been suggested that early cancer is better for studying the effects of psychosocial factors, and secondly, that some cancer types, including breast cancer, are probably influenced by psychosocial factors via the neuro-hormonal-immunological pathway (Kiecolt-Glaser and Glaser, 1995, 1999; Garssen, 2004). The variables were selected based on a large amount of previous research and a theoretical model that we had constructed earlier (Lehto *et al*, 2005). They were assessed with validated quantitative methods with good psychometric properties (Lehto-Järnstedt, 2000). Earlier studies have rarely taken all elements of psychological stress processes (Dolbeaut *et al*, 1999; Lehto *et al*, 2005) into account simultaneously (Barraclough *et al*, 1992; Petticrew *et al*, 2002; Garssen, 2004; Lehto *et al*, 2006), and have usually neglected socioecomomic status (Garssen and Goodkin, 1999). Centralised cancer care in Finland allowed us to collect a representative sample with a good participation rate. Ethnic differences, which could have influenced the psychosocial processes, hardly existed in this population from central Finland.

The strongest predictor of survival was local lymph node status, as suggested, and chemotherapy was an effective treatment. However, the effect of an important biological prognostic factor, gradus 3, vanished when the psychosocial variables were included. Two factors belonging to the multidimensional construct of socioeconomic status (Anderson and Armstead, 1995; Kristenson *et al*, 2004) predicted survival, that is, a long education predicted a favourable prognosis and unemployment predicted a shorter time without relapse. High socioeconomic status has been reported to prolong survival in cancer (Karjalainen and Pukkala, 1990; Forsen, 1991; Woods *et al*, 2006) and its effects may be mediated by psychobiological mechanisms related to stress physiology (Kristenson *et al*, 2004); people with low socioeconomic status may have more stressors and fewer resources to cope with them.

Emotional defensiveness/antiemotionality refers to a personality trait to control, suppress, or repress negative emotions in interpersonal relationships and it is one of the several concepts addressing nonexpression of negative emotions. (In behavioural medicine, ‘repression’ is often used as a synonym for nonexpression of negative emotions and the concept of consciousness is generally not included in the definition, unlike the use of the term in psychodynamic literature (Garssen and Remie, 2004)). These concepts include a variety of psychiatric and psychological constructs concerning defence mechanisms, repression, emotional nonexpression, and social and personal defensiveness (Panagopoulou *et al*, 2002; Garssen and Remie, 2004). Antiemotionality may be closest to personal defensiveness, and it is also close to the main domain of the cancer-prone Type C response style (Temoshok, 1987; Garssen and Remie, 2004), which is described by nonexpression of negative emotions, such as anger and fear (Garssen and Remie, 2004). Emotional defensiveness has been suggested as being a distinctive risk factor in cancer (Fernandez-Ballesteros *et al*, 1997). However, its definition is not clear (Garssen and Remie, 2004). In this study, the unfavourable effect of emotional defensiveness was strong and persistent. This agrees with previous findings on the favourable effect of more expression and less suppression of emotions on breast cancer progression (Reynolds *et al*, 2000) and the unfavourable effect of antiemotionality in cancers (Grossarth-Maticek *et al*, 1985; van der Ploeg *et al*, 1989). Our scale evaluated control of anger in particular (Swan *et al*, 1992), and we found it to be associated with the Anger Control trait of the highly validated AX/Scale, which was, however, not predictive on breast cancer survival (In corresponding data concerning localised melanoma (Lehto *et al*, 2006), we found Anger-in and Anger Control traits to predicted worse survival.). In contrast to other findings, Japanese male patients with dichotomously (yes/no) measured rationality/antiemotionality have been found to have a reduced risk of death from cancer (Terada *et al*, 2000; Hirokawa *et al*, 2004). This may be due to differences between the effects of various levels of the trait, cultural differences, or differences between genders or cancer sites.

As in previous research, we identified only one protective psychological factor, a coping pattern Distancing, which is close to minimising (see footnote c). Denying or minimising the fact of having the cancer has long been suggested to be a predictor of a favourable cancer prognosis (Greer *et al*, 1979, 1990; Butow *et al*, 1999; Watson *et al*, 1999; Butow *et al*, 2000; Petticrew *et al*, 2002; Garssen, 2004; Lehto *et al*, 2006). Minimising the impact of the diagnosis is milder and nowadays a more realistic form of denial (Garssen, 2004). It refers to minimising the seriousness of a medical condition (not the subsequent negative affect) (Garssen and Remie, 2004). Minimising can either be an event-driven response or reflect a habitual style of minimising unpleasant events (Garssen and Remie, 2004). In recent studies (Butow *et al*, 1999, 2000; Brown *et al*, 2000), it has been found to be associated with longer survival in advanced breast cancer and melanoma (Butow *et al*, 1999, 2000).

Behavioural Escape-Avoidance, previously found to have harmful effect on the psychosocial well-being of cancer patients (Trask *et al*, 2001; Lehto *et al*, 2005), was also associated with reduced survival (the pattern used here was, however, not identical to the one used in our QOL study (Lehto *et al*, 2005)). This pattern (footnote b) also included avoidance of other people, which may tentatively lead to a low level of social support, which is suggested to reduce survival (Maunsell *et al*, 1995), and to overeating, smoking, and drinking, which may decrease survival purely via physiological processes.

Depression has long been suspected as having an unfavourable effect on cancer progression, but the findings have remained contradictory (Kaplan and Reynolds, 1988; Garssen and Goodkin, 1999; Watson *et al*, 1999; Garssen, 2004). In our study, depressive symptoms were predictive on overall survival only when emotional defensiveness was controlled, but the coping patterns were not taken into account. It has also earlier been suggested that the effect of depression is nonindependent, that is, connected to that of the other psychosocial factors, such as nonexpression of negative emotions (Garssen and Goodkin, 1999) and helplessness/hopelessness.

The unfavourable effect of a high level of perceived support may also be due to the nonexpression of negative emotions. Support was not evaluated as the social support that was actually received (Lehto-Järnstedt *et al*, 2004) but as patients' perceptions of the support hypothetically available if needed (an intrapsychic domain). High scores in this kind of support measure may be related to over-reporting in well-being measures (In our corresponding data on localised melanoma (Lehto *et al*, 2006), we found over-positively reported QOL to be highly predictive of shorter survival.) and reflect repression of negative affect (Garssen and Remie, 2004; Myers and Derakshan, 2004). In line with a previous study (Kornblith *et al*, 2001), a large proportion (15–44%) of our patients reported the highest possible level of perceived support in the MOS Survey. It is claimed that between 10 and 20% of the population answer self-report scales on distress in an overly positive fashion (Myers and Derakshan, 2004) because they avoid negative affect, that is, they possess a repressive coping style and thus report less distress compared with non-repressors. The over-reporting of perceived support may reflect the under-recognition of needs and feelings that is included in Type C behaviour. Furthermore, without suggesting repression as explanation, it is difficult to interpret why emotional defensiveness Table 3, footnote a was associated with a high level of support. It has been claimed that exclusive reliance on standard self-report methods with global ratings is an unsatisfactory way of eliciting information from repressors (Myers and Derakshan, 2004). Using measures that require specific answers instead of global ratings may help to solve this problem. Although the MOS Survey (Sherbourne and Stewart, 1991) is highly validated and useful, it has, in this same sample, been only partly associated with the cancer-specific support actually received (Lehto-Järnstedt *et al*, 2004). The current findings suggest that this method may be insufficient for evaluating social support in (breast) cancer.

The factors we found to affect survival may be interpreted to reflect relatively stable personality-related characteristics. This finding is most probably due to using a single baseline measurement only (Segerstrom, 2003) instead of using repeated measurement of psychosocial factors and thus it does not indicate a greater importance of personality-related elements over psychosocial and stress factors.

Our findings support the idea that emotional non-expression has an outstanding adverse effect and, on the other hand, denial/minimising a favourable effect on cancer survival (Garssen, 2004). We conclude that the psychological processes affecting cancer survival comprise a combination of various interacting elements and that socioeconomic factors are of importance. Emotional nonexpression seems to be related to various concepts that are suggested to affect cancer progression, such as the Type C construct and depression. Also, the patients with high emotional nonexpression or a repressive coping style may over-report in global well-being scales. This offers an explanation for the apparently astonishing findings that good well-being predicts poorer survival. High scores in global well-being measures may refer to an additional psychosocial risk factor, which needs to be studied further and which may require clinical attention in terms of screening, care or focused intervention.

## Figures and Tables

**Table 1:** Disease and treatment variables in patients

**Variable**	**% (*n*=102)**
*Tumour size (largest diameter)*	
Range 4–80 mm, mean 17.4 (s.d. 11.7)	
	
*Stage*	
I	54
II	39
III	4
Undetermined	2
	
*Histologic type*	
Ductal carcinoma	72
Lobular carcinoma	20
	
*Grade*	
1	39
2	32
3	16
	
*Presence of hormone receptors*	
Oestrogen receptors	83
Progesterone receptors	67
Either	84
	
*Metastases*	
None	66
Regional	32
Number of positive lymph nodes: range 1–15, mean 2.88 (s.d. 2.98)	
Amount of positive lymph nodes out of the total: range 0.1–1, mean 0.39 (s.d. 0.24)	
	
*Baseline surgery*	
Breast conserving	60
Mastectomy (simple or modified radical)	39
	
*Baseline adjuvant treatment*	
Surgery only	28
+ Radiotherapy	72
+ Chemotherapy	24
+ Hormonal therapy	24
Presence of other chronic disease	44

**Table 2:** Sociodemographic and socioeconomic variables in patients

**Variable**	**% (*n*=102)**
*Age*	
Mean 54.2 (s.d. 8.45) years	
	
*Gender*	
Male	1
Female	99
	
*Marital status*	
Single	12
Married or cohabiting	68
Divorced	15
Widowed	6
	
*Level of education*	
Basic	31
Medium	31
College or higher	34
Not known	2
	
*Employment status (baseline)*	
Employed	55
Unemployed	13
Housewife	1
Retired	31

**Table 3:**
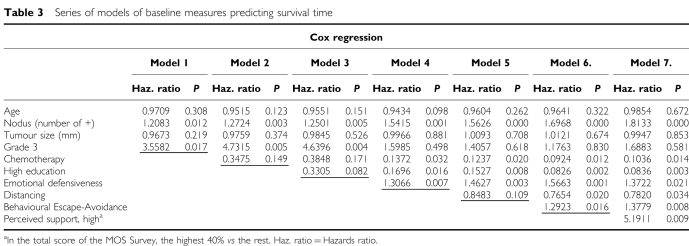
Series of models of baseline measures predicting survival time

**Table 4:** Baseline measures predicting survival in patients with no local metastases

**Cox regression**			
	**Haz. ratio**	***z***	***P*>∣*z*∣**
Age	0.9240	−1.37	0.170
Tumour size (mm)	0.9990	0.04	0.970
High education	0.0761	−2.46	0.014
Emotional defensiveness	1.6904	2.67	0.008
Distancing	0.7843	−1.50	0.135
Behavioral Escape-Avoidance	1.2840	1.83	0.068
Perceived support, high	7.4078	2.61	0.009

Haz. ratio=Hazards ratio.

**Table 5:** Baseline psychosocial measures predicting event-free time

**Cox regression**			
	**Haz. ratio**	***z***	***P*>∣*z*∣**
Age	0.9501	−1.81	0.070
Local metastases (number of +)	1.5998	4.14	0.000
Tumour size (mm)	0.9852	−0.77	0.439
Grade 3	1.3668	0.46	0.643
Chemotherapy	0.1103	−3.00	0.003
High education	0.5154	−1.43	0.153
Unemployment	2.7154	1.84	0.066
Emotional defensiveness	1.1304	1.66	0.098
Depressive symptoms	1.0674	1.84	0.066
Perceived support, high	2.1766	1.79	0.074

Haz. ratio=Hazards ratio.

## Footnotes

a Emotional defensiveness comprising, for example, items ‘If someone deeply hurts my feelings, I still try to treat them reasonably and to understand their behaviour…’, ‘I try to understand people even if I do not like them…’, If someone acts against my needs and desires, I still try to understand him/her…’

b Coping pattern Behavioural Escape-Avoidance included, for example, items: I generally avoided being with people; I tried to make myself feel better by eating, drinking, smoking, using drugs, or medications; I took a big chance or did something very risky to solve the problem; I took it out on other people….

c Coping pattern Distancing included, for example, items: Tried to keep my feelings from interfering . . .; Didn't let it get to me, refused to think about it; Made light of it, refused to get too serious; Went on as if it were not happening; Tried to keep my feelings to myself; Looked for a silver lining, looked on the bright side….
